# COVID-19 pneumonia: phenotype assessment requires bedside tools

**DOI:** 10.1186/s13054-020-02973-9

**Published:** 2020-05-29

**Authors:** Zhanqi Zhao, Wan-Hsuan Kung, Hou-Tai Chang, Yeong-Long Hsu, Inéz Frerichs

**Affiliations:** 1 Department of Biomedical Engineering, Fourth Military Medical University, Xi’an, China; 2 Institute of Technical Medicine, Furtwangen University, Villingen-Schwenningen, Germany; 3 Division of Chest Medicine, Far Eastern Memorial Hospital, Taipei, Taiwan; 4 Department of Critical Care Medicine, Far Eastern Memorial Hospital, No. 21, Sec. 2, Nanya S. Rd., Banciao Dist., New Taipei City, 220 Taiwan; 5 Department of Anesthesiology and Intensive Care Medicine, University Medical Center of Schleswig-Holstein, Campus Kiel, Kiel, Germany

**Keywords:** Covid-19, ARDS, Mechanical ventilation

Dear Editor,

We read with interest the Editorial by Gattinoni et al. proposing two phenotypes for COVID-19 pneumonia and the corresponding respiratory treatments [1]. Type 1 is characterized with high compliance, low ventilation-to-perfusion ratio and low lung recruitablity. Type 2 is with low compliance and high lung recruitability. We appreciate the effort to classify COVID-19 into phenotypes and to propose the corresponding respiratory treatments. We would like to point out that another phenotype is often presented in COVID-19-associated moderate to severe ARDS, based on our observation and discussions with colleagues treating these patients.

Different from the phenotypes described in [1], the COVID-19 patients we encountered had rather low compliance and their lungs were non-recruitable, despite of large amount of non-aerated tissue. When assessing the lung recruitability with either the bedside estimates suggested in [2], or with electrical impedance tomography (EIT) [3, 4], we found that instead of recruiting non-aerated lung tissue, increasing PEEP to around 15 cmH_2_O rather induced overdistension in previously ventilated regions. The finding was coincided with the results of a recent study where the majority of the reported patients were poorly recruitable with high PEEP even though the compliance was fairly low [5].

Figure 1 shows EIT measurement of a COVID-19 patient during PEEP increase from 8 to 16 cmH_2_O, and the chest X-Ray on the same day. The patient was ventilated under volume-controlled mode. Respiratory system compliance slightly decreased from 23 to 22 ml/cmH_2_O. Overdistension was observed in non-dependent regions compared to the lower PEEP (orange regions in Fig. 1c). No recruitment was found in dependent regions. Since the tidal volume was fixed (6 ml/kg predicted body weight), ventilation was redistributed from overdistended regions to other open regions (blue regions in Fig. 1c). In such case, although the compliance was low, high PEEP would not recruit lung tissues but rather pose a risk of barotrauma.

**Fig. 1:**
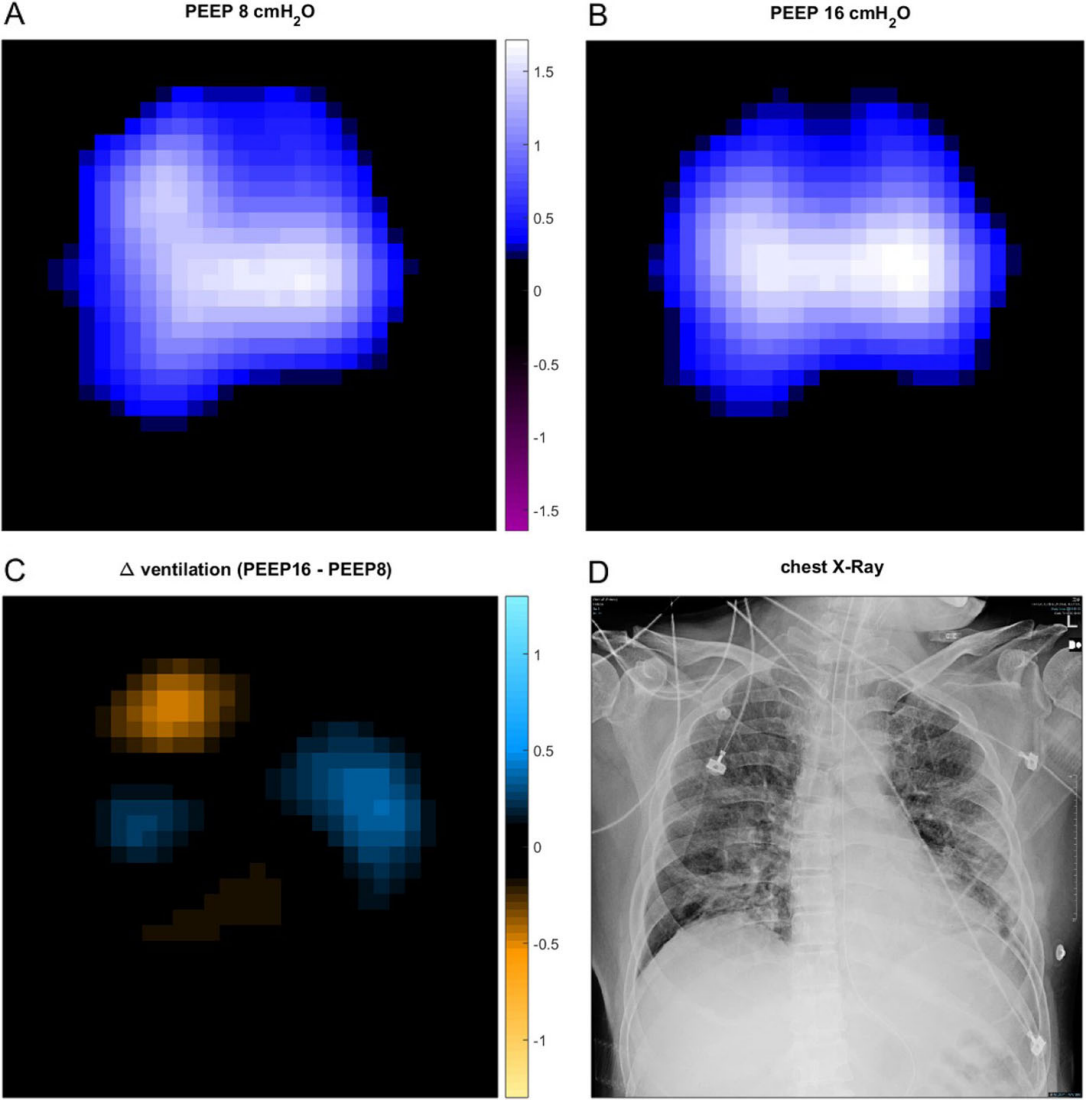
Electrical impedance tomography measurement of a COVID-19 patient during PEEP increases from 8 to 16 cmH_2_O, and the chest X-ray on the same day. **a, b** Tidal variations show ventilation distribution during tidal breathing. Highly ventilated regions are marked in light blue. **c** The differences in ventilation distribution between PEEP of 16 and PEEP of 8 cmH_2_O. Ventilation loss is marked in orange whereas ventilation gain is marked in blue. **d** Chest X-ray shows increased infiltration to the left lower lung field and to the right lower lobe

The disease status of COVID-19 patients developed rapidly. As pointed out in [1], CT could be the best way to identify the phenotypes; however, it might not be practical due to the overwhelming number of patients. Besides, the SARS-CoV-2 virus is highly infectious, which makes the transportation of patients for CT examination very difficult. Bedside tools such as EIT and ultrasound may play an important role in identifying different phenotypes for COVID-19 patients. In addition, such functional tools permit monitoring of the patients’ response to various therapeutic interventions, which in turn helps guiding treatments.

## Authors’ response

### Atypical, early, and late ARDS: the evolution of COVID-19 pneumonia

Luciano Gattinoni, Davide Chiumello, Sandra Rossi

We thank Zhao [6] and coworkers for their interest in our editorial [1]. We proposed two phenotypes (types 1 and 2, that we later called L and H) as a two “extremes” of a spectrum of respiratory failure in COVID-19 pneumonia. What for us was more striking was the remarkable dissociation between compliance and hypoxemia in L patients [7], when some of them, because of either the natural progression of the disease or the lack of prevention of possible patient self-inflicted lung injury, shift to the Type H, which qualifies as typical ARDS. What Zaho et al. added to this framework is the possibility of a further progression of the disease to fibrotic state, which we also observed in type 2 COVID-19 patients in late stages (more than 1 week), if unable to heal from the disease [8]. Shifting from prevalent edema to prevalent fibrosis is characterized by a progressive reduction of response to PEEP. Unfortunately, the prevalent fibrosis typical of the later stage, instead of prevalent edema, cannot be easily detected by imaging, but it is associated with a progressive deterioration of lung mechanics and PaCO_2_ rise, associated to severe structural damage of the lung [9]. What is important to realize, however, in this disease is that the mechanism of hypoxemia and the respiratory treatment in the type 1 early phase are different from typical ARDS. The type 2, if unsolved, with time shifts, as observed by our colleagues in their correspondence, to a fibrotic status, typical of late ARDS.

## Data Availability

Not applicable.
